# P-1301. Effective Durations of Antibiotic Therapy for Stenotrophomonas maltophilia Bloodstream Infections

**DOI:** 10.1093/ofid/ofaf695.1489

**Published:** 2026-01-11

**Authors:** Christina Guo, Justin Suero, Pranita Tamma

**Affiliations:** Johns Hopkins Bloomberg School of Public Health, Beaumaris, Victoria, Australia; Johns Hopkins Hospital, Baltimore, Maryland; Johns Hopkins University School of Medicine, Baltimore, Maryland

## Abstract

**Background:**

*Stenotrophomonas maltophilia* is an increasingly important cause of healthcare-associated bloodstream infections (BSI), but optimal treatment duration remains unclear. Clinical trials supporting 7-day therapy for uncomplicated gram-negative BSI included few or no *S. maltophilia* cases, limiting applicability. This study compared outcomes between short- and prolonged-course therapy for *S. maltophilia* BSI.

**Figure:**
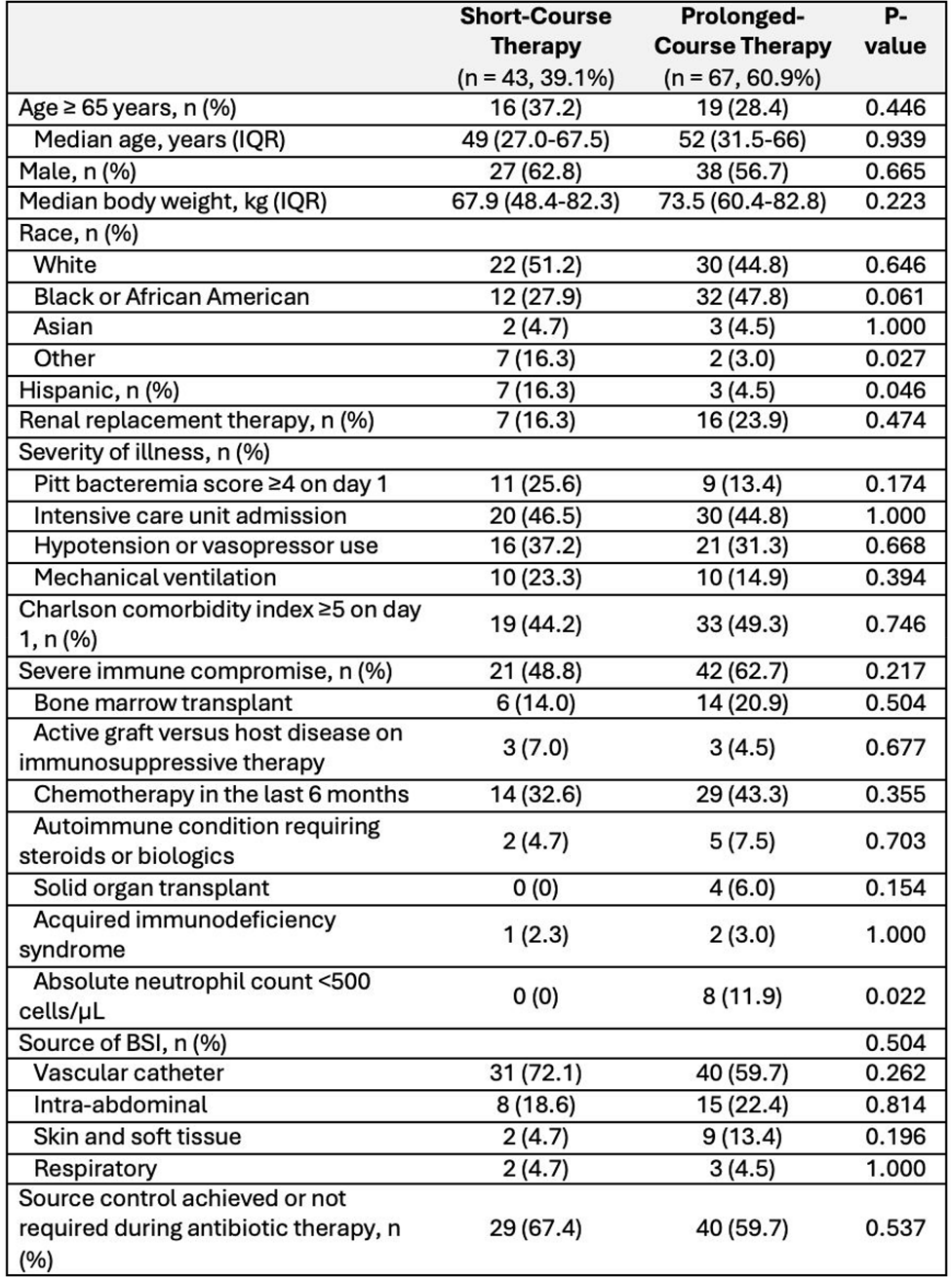
Baseline characteristics of the unweighted cohort

**Figure:**
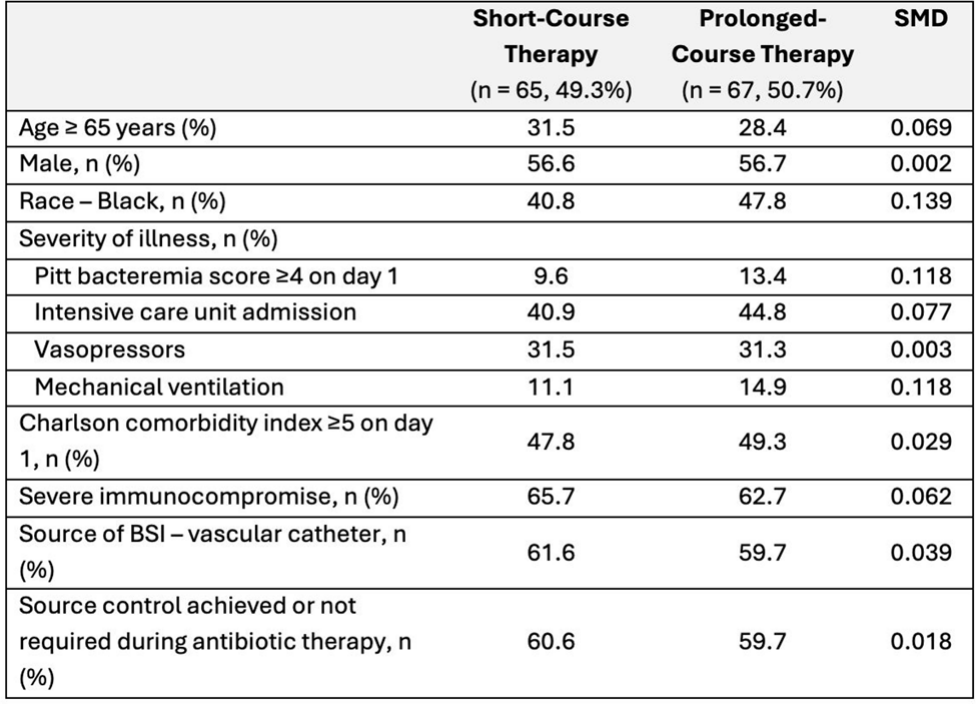
Baseline characteristics of the IPTW cohort

**Methods:**

A retrospective cohort study was conducted among patients with *S. maltophilia* BSI treated with 7-21 days of active antibiotic therapy within The Johns Hopkins Hospital System from 2018 to 2024. Patients were stratified into short-course (7-11 days) or prolonged-course (12-21 days) groups. The primary outcome was 30-day all-cause mortality. Secondary outcomes included recurrence within 90 days, infection-related hospital length of stay, and antibiotic-associated adverse events. Inverse probability of treatment weighting (IPTW) was used to limit confounding by indication and weighted logistic regression was used to estimate associations between treatment duration and outcomes in the IPTW cohort.

**Figure:**
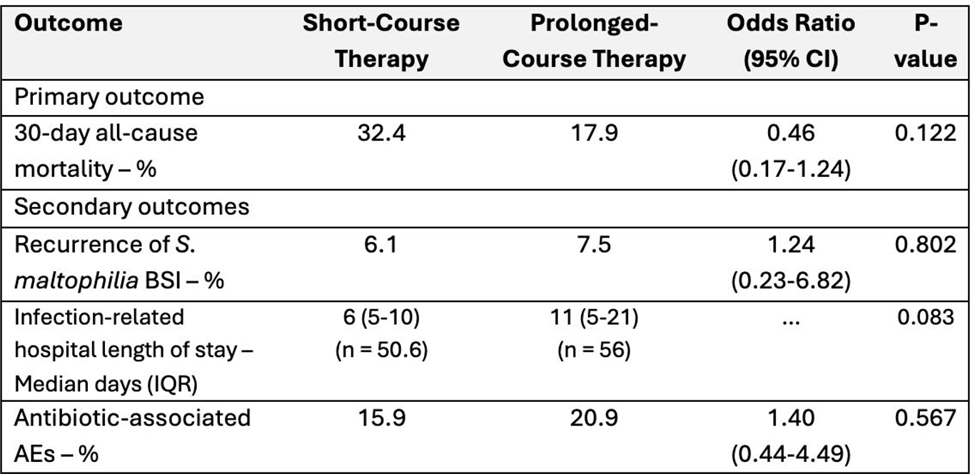
Outcomes of short-course vs prolonged-course antibiotic therapy in S. maltophilia BSI in the IPTW cohort

**Results:**

Among 110 patients treated for *S. maltophilia* BSI, 43 (39%) received short-course and 67 (61%) prolonged-course therapy. Median treatment durations were 9 days (IQR 8-10) and 16 days (IQR 14-17) for short- and prolonged-course respectively. Approximately 45% of patients required intensive care, 47% had a Charlson Comorbidity Index ≥4, and 57% had severe immune compromise. In the IPTW cohort, 30-day mortality was 32% vs 18% in the short- vs prolonged-course groups. Although not meeting statistical significance, prolonged-course therapy was associated with lower odds of 30-day mortality compared to short-course therapy (OR 0.46, 95% CI: 0.17-1.24; p = 0.12). There were no differences in recurrence (6% vs. 8%; p = 0.80) or antibiotic-associated adverse events (16% vs. 21%; p = 0.57). There was a non-significant trend toward shorter infection-related stay in the short-course group (6 [IQR 5-10] vs. 11 [IQR 5-21] days; p = 0.08).

**Conclusion:**

Although not statistically significant, numerical differences in mortality suggest further studies are needed to ensure that short-course therapy is as effective as more prolonged durations for *S. maltophilia* BSI.

**Disclosures:**

All Authors: No reported disclosures

